# Imaging findings in patients with axial spondyloarthritis presenting with recurrent fever attacks: data from the international AIDA network spondyloarthritis registry

**DOI:** 10.3389/fmed.2025.1662890

**Published:** 2025-09-15

**Authors:** Nunzia Di Meglio, Cristian Sica, Antonio Vitale, Valeria Caggiano, Armando Perrella, Vito Di Martino, Margherita Di Stefano, Mauro Fratarcangeli, Giulio Bagnacci, Piero Ruscitti, Paola Cipriani, Azadeh Shariat Panahi, Samar Tharwat, Hanan M. Elberashi, Esraa E. Othman, Alessandro Conforti, Linda Lucchetti, Fabrizio Varini, Jurgen Sota, Guillermo Arturo Guaracha-Basañez, Perla Ayumi Kawakami-Campos, Eduardo Martín-Nares, Maissa Thabet, Ben Yahia Wissal, Sukran Erten, Mehmet Akif Eksin, Bahar Özdemir Ulusoy, Patrizia Barone, Santi Papa, Andrea Giugno, Stefano Gentileschi, Carla Gaggiano, Simona Buonanno, Soad Hashad, Halah Etayari, Abdurrahman Tufan, Gaafar Ragab, Ewa Wiesik-Szewczyk, José Hernández-Rodríguez, Alberto Balistreri, Andrea Hinojosa-Azaola, Claudia Fabiani, Bruno Frediani, Luca Cantarini, Maria Antonietta Mazzei

**Affiliations:** ^1^Unit of Diagnostic Imaging, Department of Medical, Surgical and Neuro Sciences and of Radiological Sciences, University of Siena, Azienda Ospedaliero-Universitaria Senese, Siena, Italy; ^2^Azienda Ospedaliero-Universitaria Senese [European Reference Network (ERN) for Rare Immunodeficiency, Autoinflammatory and Autoimmune Diseases (RITA) Center], Siena, Italy; ^3^Department of Medical Sciences, Surgery and Neurosciences, Research Center of Systemic Autoinflammatory Diseases and Behçet’s Disease Clinic, University of Siena, Siena, Italy; ^4^Rheumatology Unit, Department of Biotechnological and Applied Clinical Sciences, University of L’Aquila, L’Aquila, Italy; ^5^Rheumatology and Immunology Unit, Internal Medicine Department, Mansoura University, Mansoura, Egypt; ^6^Department of Internal Medicine, Faculty of Medicine, Horus University, New Damietta, Egypt; ^7^General Medicine Unit, San Paolo di Civitavecchia Hospital, Civitavecchia, Italy; ^8^Hospital Pharmacy Unit, San Paolo di Civitavecchia Hospital, Civitavecchia, Italy; ^9^Laboratory of Experimental Oceanology Marine Ecology, Department of Ecological and Biological Sciences (DEB), University of Tuscia, Civitavecchia, Italy; ^10^Department of Immunology and Rheumatology, Instituto Nacional de Ciencias Médicas y Nutrición Salvador Zubirán, Mexico City, Mexico; ^11^Department of Ophthalmology, Instituto Nacional de Ciencias Médicas y Nutrición Salvador Zubirán, Mexico City, Mexico; ^12^Internal Medicine Department, Farhat Hached University Hospital, Faculty of Medicine of Sousse, University of Sousse, Sousse, Tunisia; ^13^Department of Rheumatology, Faculty of Medicine, Ankara City Hospital, Ankara Yıldırım Beyazıt University, Ankara, Türkiye; ^14^Clinic of Rheumatology, Ankara Gaziler Physical Therapy and Rehabilitation Training and Research Hospital, Ankara, Türkiye; ^15^Pediatric Rheumatology Unit, Department of Integrated Maternal-Child and Reproduction Activity, AOU Policlinico-San Marco, Catania, Italy; ^16^Rheumatology Department, Tripoli Children’s Hospital, Omar Almukthar Street, Tripoli, Libya; ^17^Department of Internal Medicine, Division of Rheumatology, Gazi University Hospital, Ankara, Türkiye; ^18^Rheumatology and Clinical Immunology Unit, Internal Medicine Department, Faculty of Medicine, Cairo University, Giza, Egypt; ^19^Faculty of Medicine, Newgiza University, 6th of October City, Egypt; ^20^Department of Internal Medicine, Pneumonology, Allergology, Clinical Immunology, and Rare Diseases, Military Institute of Medicine, National Research Institute, Warsaw, Poland; ^21^Clinical Unit of Autoinflammatory Diseases, Department of Autoimmune Diseases, Institut d’Investigacions Biomèdiques August Pi I Sunyer (IDIBAPS), Hospital Clínic of Barcelona [European Reference Network (ERN) for Rare Immunodeficiency, Autoinflammatory and Autoimmune Diseases (RITA) Center], University of Barcelona, Barcelona, Spain; ^22^Bioengineering and Biomedical Data Science Lab, Department of Medical Biotechnologies, University of Siena, Siena, Italy; ^23^Ophthalmology Unit, Department of Medicine, Surgery and Neurosciences, University of Siena, Siena, Italy

**Keywords:** spondyloarthritis, autoinflammatory disease, fever attacks, magnetic resonance imaging, sacroiliitis, power doppler ultrasound

## Abstract

Spondyloarthritis (SpA) is a group of immuno-mediated diseases likely caused by a complex interaction between autoimmune and autoinflammatory immunological mechanisms, where a febrile clinical presentation can be an early manifestation. This retrospective study aims to assess the prevalence of inflammatory involvement of the sacroiliac joints (SIJs), pelvis, and lumbosacral spine in patients with axial-SpA and recurrent febrile presentation. MR examinations of 57 patients fulfilling the axial-SpA according to ASAS criteria and presenting with febrile symptoms were evaluated, compared to 30 patients with axial-SpA and no febrile symptoms. 20/57 patients in the axial-SpA group with recurrent fevers underwent a US examination of the SIJs. Structural damage and inflammatory alterations of the SIJs were highly prevalent in both groups. In patients with febrile syndrome, bone marrow edema (78.9%) and erosions (85.9%) were the most prevalent findings in the SIJs; SPARCC score was significantly higher in patients with typical axial-SpA onset (BME: 20.1 ± 12.28 vs. 6.15 ± 3.21; erosions: 22.16 ± 8.13 vs. 6.60 ± 3.08; *p* = 0.01). Among pelvic enthesitis, enthesitis of the pubic symphysis showed a significant difference in prevalence (*p* < 0.001). Significant differences were found for the prevalence of vertebral body corner sclerosis (*p* = 0.01), zygapophyseal capsulitis (*p* = 0.005), and interspinous enthesitis (*p* = 0.006). No significant correlation was found between ultrasound findings and MR inflammatory changes (*p* > 0.05). SIJs and spinal inflammatory alterations and pelvis enthesitis were highly prevalent in axial-SpA patients with and without recurrent fever. Enthesitis of the pubic symphysis, vertebral body corner sclerosis, zygapophyseal capsulitis, and interspinous enthesitis showed a significant difference in frequency between axial-SpA patients with and without fever attacks.

## Highlights

- Axial spondyloarthritis (SpA) may be associated with extra-articular inflammatory manifestations, including periodic febrile episodes. Febrile syndromes indicate mixed pathogenesis, autoimmune and autoinflammatory in axial SpA.
- The radiological features of spondyloarthritis identified in patients with febrile episodes are largely comparable to those of patients without febrile episodes. However, the latter tend to exhibit a greater frequency of enthesitis of the pubic symphysis, vertebral body corner sclerosis, zygapophyseal capsulitis, and interspinous enthesitis.
- Clinical statement: Patients with axial SpA and febrile presentation have a high incidence of inflammatory changes of the sacroiliac joints (SIJs), pelvis and lumbosacral spine on MR. The most commonly reported changes are subchondral bone marrow edema and joint erosions in SIJs.

## Introduction

Axial spondyloarthritis (SpA) represents a group of chronic inflammatory joint disorders characterized by the axial skeleton’s involvement, with or without peripheral joints (1). Axial SpA encompasses a range of diseases, including ankylosing spondylitis, psoriatic arthritis, arthritis associated with inflammatory bowel diseases, reactive arthritis, and undifferentiated arthritis. It represents the second most prevalent form of chronic inflammatory arthritis, with an estimated prevalence of 0.5–1.5% in the Caucasian population (2). In addition to joint symptoms, axial SpA has been associated with extra-articular inflammatory manifestations such as cutaneous psoriasis, uveitis, or chronic inflammatory bowel diseases, including Crohn’s disease or ulcerative colitis (2).

Axial SpA has long been considered an immunological disorder with a mixed pattern of autoimmune and autoinflammatory disease (3). However, a greater role for autoinflammatory pathogenesis has recently been suggested by the emerging predominant involvement of innate immunity (4, 5): it is established that, although infrequently, axial SpA can present with febrile episodes, and fever is considered one of the most common symptoms of autoinflammatory disorders (6, 7). Currently, there is still limited data characterizing the subgroup of patients with axial SpA and recurrent febrile episodes, and this remains a subject of investigation from clinical, pathogenetic, and also radiological perspectives. Therefore, the present study was conducted to achieve a better knowledge of the magnetic resonance (MR) and ultrasound features of patients suffering from axial SpA and recurrent febrile attacks.

## Materials and methods

### Study design

The primary objective of this study was to retrospectively investigate the pattern and prevalence of MR and ultrasound features of axial SpA in a cohort of patients presenting with apparently unexplained recurrent fever, in whom axial SpA fulfilled the Assessment of SpondyloArthritis international Society (ASAS) criteria during the diagnostic workup (8, 9). An additional objective was to assess any correlation between ultrasound findings and MR-detected active inflammatory changes.

From April 2021 to November 2022, we prospectively identified and enrolled 57 patients (39F, 18 M, median age 42 years, IQR 14–75). The data were collected through the International AutoInflammatory Disease Alliance (AIDA) Network registry for Undifferentiated Systemic AutoInflammatory Diseases (USAIDs) (10, 11). This registry functions as an observational study, intending to compile information from patients receiving treatment in accordance with the established standard of care. All patients were newly diagnosed with non-radiographic axial SpA by an expert rheumatologist based on clinical assessment and the subsequent application of classification criteria, as part of the diagnostic process for undiagnosed fever. Patients included in the study had previously undergone X-rays of the pelvis and entire spine to exclude those with radiographic axial SpA.

Patients reported inflammatory low back pain with or without other extra-articular manifestations associated with axial SpA, and at least one not otherwise explainable febrile episode per year, evaluated and confirmed by physicians as a fever of no apparent cause. Subsequent magnetic resonance (MR) imaging of SIJs confirmed the presence of radiologic signs consistent with axial SpA: ASAS criteria (imaging arm) were met in all cases (8, 9, 12, 13). The control group comprised 30 patients (20F, 10 M, median age 41.5 years, IQR 16–70) with a new diagnosis of axial SpA with chronic inflammatory low back pain and no unexplained fever attacks. All patients were Caucasian and were not undergoing any specific therapy.

Exclusion criteria encompassed reactive SpA, infections, autoimmune and neoplastic diseases, and gastrointestinal and/or urinary infections in the 6 months before the onset of symptoms. Radiographic axial SpA were excluded *a priori* from the control group, as they were not present in the axial SpA group with febrile episodes. Inclusion criteria required the fulfillment of ASAS criteria, irrespective of the presence of recurrent fever attacks, along with the provision of informed consent to participate in the project, which was obtained from all patients, parents, or legal guardians (10, 11). The study also included patients under 16 years of age diagnosed with juvenile idiopathic arthritis involving the sacroiliac joints.

Clinical characteristics of patients included in this study are reported in Table 1.

**Table 1:** Demographic characteristics of patients and frequency of the specific clinical and laboratory item included in the assessment of spondyloarthritis international society (ASAS) criteria.

Clinical item	Axial SpA with recurrent fever	Typical onset Axial SpA without recurrent fever
	*n* = 57	*n* = 30
Sex^a^		
Male	18 (31.5)	10 (33.3)
Female	39 (68.5)	20 (66.7)
Age		
Mean	42	41.5
Median	37	52.7
Range	14–75	16–70
Articular disease duration, years		
Mean	5.21	11.8
Median	1.7	7.9
Range	0.3–36.6	0.3–61.6
Items of ASAS criteria^a^		
Inflammatory low back pain	57 (100)	30 (100)
Arthritis	23 (40.3)	17 (56.7)
Enthesitis (heel)	4 (7)	3 (10)
Uveitis	7 (12.2)	4 (13.4)
Dactylitis	5 (9)	2 (6.7)
Psoriasis	5 (9)	12 (40)
Inflammatory Bowel Diseases	3 (5.2)	6 (20)
Response to NSAIDs^b^	27 (47.3)	19 (63.4)
Family history of SpA	9 (15.8)	10 (33.3)
Elevated C-reactive protein^c^	32 (56.1)	16 (53.3)
HLA-B27 positivity	5 (9)	7 (23.3)

SpA: spondyloarthritis; NSAIDs: non-steroidal anti-inflammatory drugs; HLA: human leukocyte antigen.

^a^Data are the number of patients with the percentage in parentheses.

^b^All the patients treated with NSAIDs experienced at least a partial improvement in the low back pain.

^c^C reactive protein assessed outside fever bouts.

The study received approval from the Ethics Committee of Azienda Ospedaliero Universitaria Senese, Siena, Italy (AIDA Project; Ref. N. 14951) as part of the AIDA Program (10, 11). The study protocol adhered to the principles of the Declaration of Helsinki.

### MR evaluation

All patients underwent an MR examination on a 1.5 T scanner (Signa Twin Speed Hdxt; GE Healthcare, USA) with a dedicated protocol for the study of SIJs and hips. The acquired sequences included a coronal “panoramic” short-time inversion recovery (STIR) sequence for hip assessment, followed by sequences with a reduced field of view (FOV) for a detailed study of the SIJs. These sequences were obtained on the oblique coronal plane, parallel to the major axis of the sacrum, and on the oblique axial plane, perpendicular to the major axis of the sacrum, according to the European Society of Musculoskeletal Radiology (ESSR) recommendation (14). Both T1-weighted and STIR sequences were utilized. In 53 out of 87 cases (60.2%), the examination was extended by acquiring a T1 gradient-echo sequence with fat suppression on the coronal oblique plane (3D spoiled Gradient-Echo T1W with spectral inversion at lipidi, FSPGR, GE Healthcare) before and after intravenous contrast media (CM) administration (Gadoterate meglumine 0.2 mg/kg). The objective of this dynamic acquisition was to obtain post-contrast images of the SIJs, followed by a volumetric panoramic coronal acquisition of the pelvis and lumbar/sacral spine, in order to gather post-contrast information on the entheses and lumbar/sacral spine. Technical parameters of the MR protocol are detailed in Supplementary Table S1.

The MR analysis was retrospectively evaluated in agreement by two readers, a radiologist with expertise in rheumatological pathology, and a senior radiology trainee. Concerning SIJs, the analysis focused on identifying signs of acute and chronic inflammatory involvement according to criteria defined by the ASAS (12, 13). Acute inflammatory findings included subchondral bone edema (BME), enthesitis, capsulitis, and joint space hyperintensity. Structural lesions included joint erosions, subchondral bone sclerosis and fat metaplasia, backfill (the presence of fat metaplasia in joint erosions), bone bud, and joint ankylosis. Furthermore, signs of enthesitis in the pelvis (ischial tuberosities, pubic symphysis, greater trochanters) were investigated. The lumbar spine included in the FOV was assessed, considering acute or chronic lesions of the vertebral body (edema, fat metaplasia, and sclerosis of vertebral body corners, i.e., Romanus lesions, presence/absence of aseptic spondylodiscitis, i.e., Andersson lesions, enthesitis of the annulus fibrosus/syndesmophytes) and posterior elements (capsulitis/enthesitis of zygapophyseal joints and enthesitis of interspinous and supraspinous ligaments). BME in the SIJs was quantified using the MR score proposed by the Spondyloarthritis Research Consortium of Canada (SPARCC) (15). A score equal to or greater than 2 was considered the minimum indicative value of active sacroiliitis on MR, with a maximum score of 72. The SPARCC score adapted for structural lesions was also utilized to assess subchondral fat metaplasia, erosions, and subchondral bone sclerosis, with a maximum score of 40 (16).

### Ultrasound evaluation

Twenty out of 57 patients in the axial SpA group with recurrent fevers had randomly undergone an ultrasound (US) examination of the SIJs. The patient was in the prone position, and the probe was positioned in a transverse scan over the median line of the sacrum, at the spinous apophysis of the first sacral vertebra. Subsequently, the probe was shifted over the top of the left first sacral foramen up to the margin of the ilium, where the probe was placed in an oblique position and the sound beam inclined about 20° downwards. The process was then repeated ab initio for the right SIJ. The first sacral foramen was always observed to avoid measurement of the pre-sacral arteries and veins, and all extensions of the SIJs were explored up to the second sacral foramen level. Any PW Doppler signal detected in the SIJs space was scored using a 3-point scale: 0 = absence of signal, 1 = isolated vessel, 2 = more than one vessel. The same vessels were also evaluated using quantitative PW Doppler, calculating the Resistive Index (RI = peak of systolic flow − end diastolic flow/peak systolic flow). At least two measurements were obtained for each vessel, and the mean value was recorded. An RI value of <0.60 was considered to be indicative of sacroiliitis, as previously performed in other studies (17–19).

### Statistics

Descriptive statistics methods were applied for the analysis of MR and US data. Statistical analyses were performed using non-parametric tests. The differences in the two study populations were analysed by applying the Chi2-test and Mann–Whitney U-test. Q-Q, and p–p plots were used to assess the normal distribution of the variables. Spearman’s Rho correlation coefficient was used to determine the correlation between quantitative variables with a non-normal distribution, and Pearson’s correlation coefficient for variables with a normal distribution. A *p* < 0.05 was considered statistically significant. All statistical analyses were performed in IBM Statistical Package for the Social Sciences (SPSS) version 22.0.

## Results

**Figure 1:**
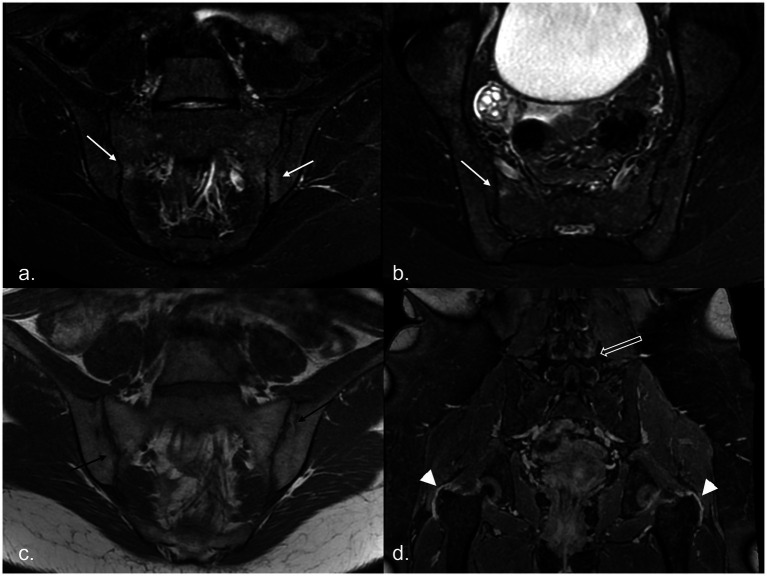
MR examination of a 20-year-old woman with recurrent fever: it shows areas of subchondral BME on the right sacral and left iliac sides of the sacroiliac joints [white arrows, panel **(a)** in the oblique coronal plane and panel **(b)** in the oblique axial plane], associated with multiple bilateral joint erosions [black arrows in panel **(c)**]. After administration of CM, in the 3D panoramic sequence for the hip, trochanteric enthesitis (arrowhead) and facet capsulitis (empty white arrow) are evident [panel **(d)**]. BME, bone marrow edema; CM, contrast media.

A comparison of MR characteristics of the axial SpA patients with recurrent fever and controls is presented in Tables 2–4. Structural damage and inflammatory alterations of SIJs were present in a large percentage of patients with both recurrent febrile presentation and typical presentation, although the prevalence was higher in the latter group (Figure 1).

**Table 2:** Frequency of acute and chronic inflammatory MR changes of the SIJs.

MR findings	Axial SpA with recurrent fever	Typical onset Axial SpA without recurrent fever	*p*
BME	45 (78.9)	30 (100)	0.007
SIJs Enthesitis	38 (66.6)	26 (86.7)	0.057
SIJs Capsulitis	32 (56.1)	23 (76.7)	0.072
Joint space hyperintensity	10 (17.5)	9 (30)	0.181
Erosions	49 (85.9)	30 (100)	0.03
Subchondral bone sclerosis	29 (50.9)	26 (86.7)	0.01
Subchondral fat metaplasia	40 (70.1)	29 (96.7)	0.004
Backfill	7 (12.3)	19 (63.3)	<0.001
Bone Bud	7 (12.3)	6 (20)	0.337
Joint ankylosis	1 (1.7)	3 (10)	0.081

BME, bone marrow edema; SIJs, sacroiliac joints; MR, Magnetic Resoncance; SpA, spondyloarthritis.

Data are the number of patients with the percentage in parentheses.

**Table 3:** Frequency of pelvic enthesitis at magnetic resonance examination.

Enthesitis site	Axial SpA with recurrent fever	Typical onset Axial SpA without recurrent fever	*p*
Greater trochanters	42 (73.7)	27 (90)	0.96
Ischial tuberosities	23 (40.4)	17 (56.7)	0.167
Pubic symphysis	4 (7)	14 (46.7)	<0.001

SpA, spondyloarthritis.

Data are the number of patients with the percentage in parentheses.

**Table 4:** Frequency of acute and chronic inflammatory changes of the spine at magnetic resonance examination.

MR findings	Axial SpA with recurrent fever	Typical onset Axial SpA without recurrent fever	*p*
Vertebral body corner BME	7 (12.3)	11 (36.7)	0.08
Andersson lesion	5 (8.7)	7 (23.4)	0.061
Vertebral body corner sclerosis	4 (7)	11 (36.7)	<0.01
Vertebral body corner fat metaplasia	1 (1.7)	2 (6.7)	0.41
Enthesitis of the annulus fibrosus/syndesmophytes	2 (3.5)	3 (10)	0.261
Zygapophyseal joints capsulitis	36 (63.1)	23 (86.7)	0.005
Interspinous/supraspinous enthesitis	41 (71.9)	29 (96.7)	0.006

MR, Magnetic Resonance; SpA, spondyloarthritis; BME, bone marrow edema. Data are the number of patients with the percentage in parentheses.

**Figure 2:**
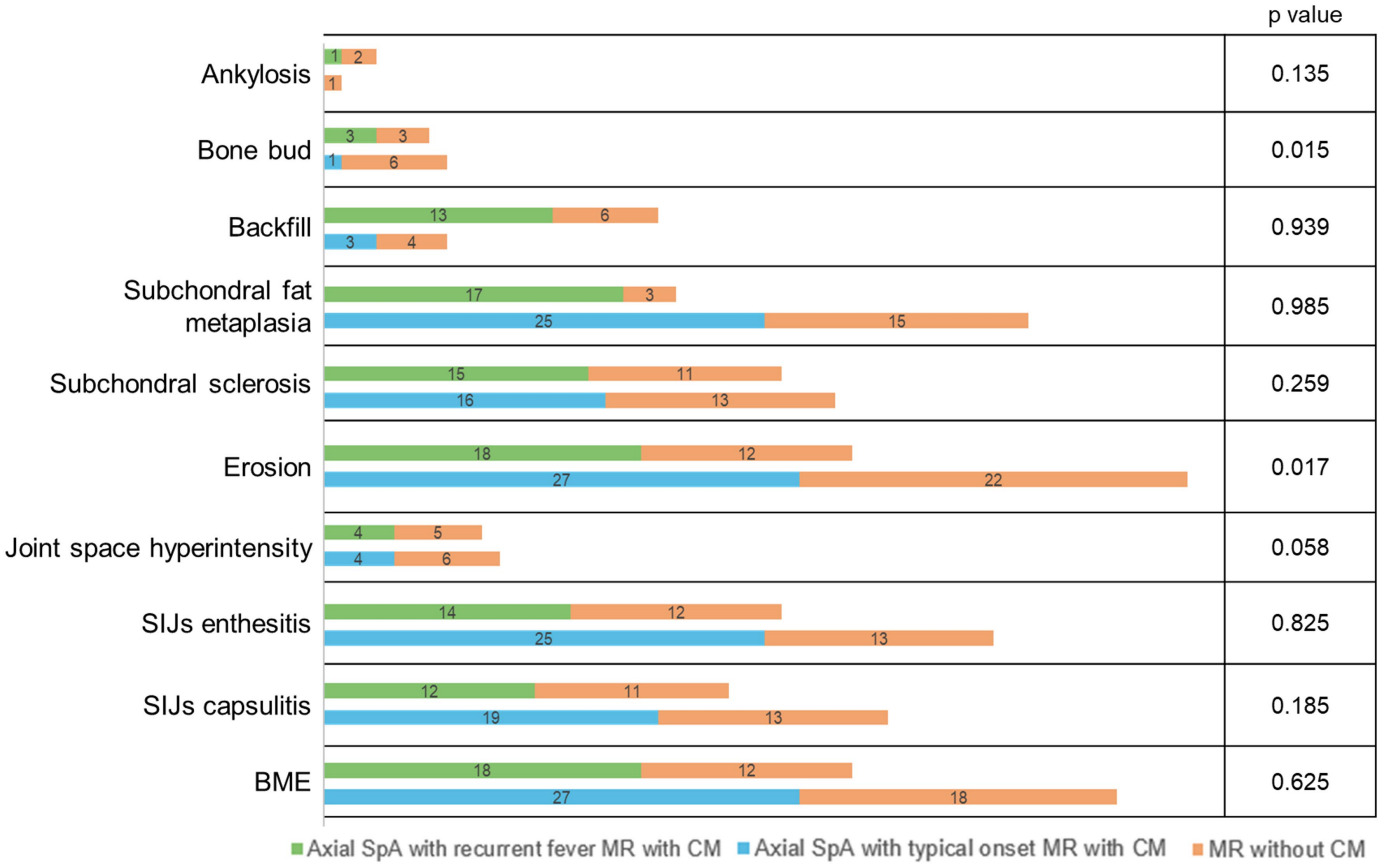
Number of patients for MR findings concerning the use of CM in axial SpA with recurrent fever and axial SpA with typical onset, respectively. *p*-values indicate the correlation between CM use and MR findings in the total population. SpA, spondyloarthritis; SIJs, sacroiliac joints; BME, bone marrow edema; CM, contrast media.

In patients presenting with febrile syndrome, 41 of 57 (71.9%) had inflammatory and structural changes of the SIJs, 5 of 57 (9%) had inflammatory changes only, and 11 of 57 (19.1%) had structural changes only. Of the 46 patients with active inflammation, 45 exhibited BME, while one showed other signs of active disease on contrast-enhanced imaging (joint space enhancement, capsulitis, and synovitis) alongside structural damage (erosions and sclerosis). In the remaining 11 patients without inflammatory changes, structural damage highly suggestive of inflammatory sacroiliitis (ankylosis, bone bud formation, marked sclerosis, and erosions) was observed. Among enthesitis of the pelvis, only enthesitis of the pubic symphysis showed a significant difference in prevalence (*p* < 0.001), with a lower frequency among patients without fever attacks. The only significant difference in the prevalence of spinal involvement was for sclerosis of vertebral body corners (*p* = 0.01), zygapophyseal capsulitis (*p* = 0.005), and interspinous enthesitis (*p* = 0.006), which were significantly more frequent among axial SpA patients without fever attacks. The number of MR changes observed with the use of CM is shown in Figure 2: a significant negative correlation with CM use, even if weak, was found only for SIJs erosions (Spearman’s Rho −0.255, *p* = 0.017), bone bud (Spearman’s Rho −0.259, *p* = 0.015), and interspinous enthesitis (Spearman’s Rho −0.276, *p* = 0.010); a negative correlation toward significance with CM use was found for joint space hyperintensity (Spearman’s Rho −0.204, *p* = 0.058).

**Figure 3:**
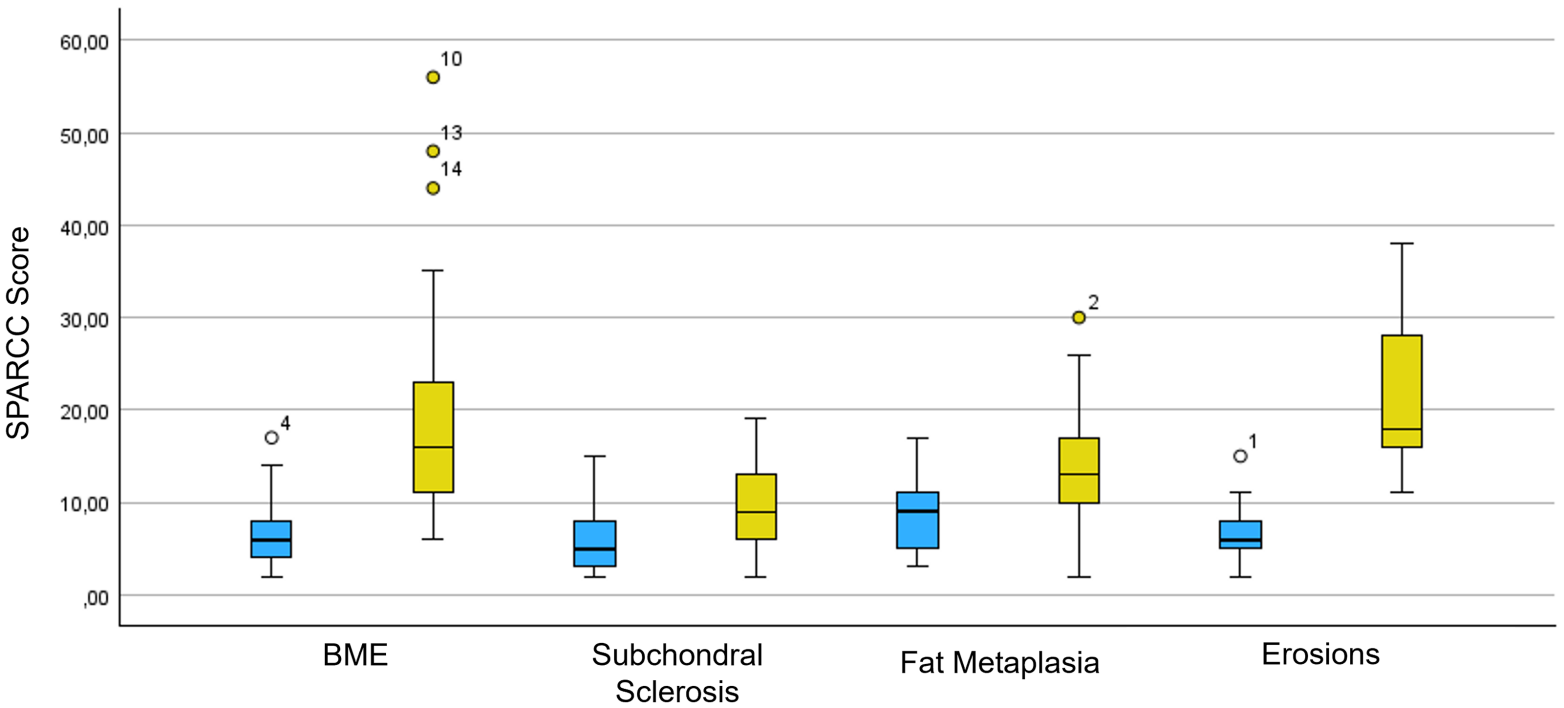
SPARCC score box plot for acute and chronic inflammatory MR changes of the SIJs. In blue is the population of axial SpA patients with recurrent fever syndromes and in yellow is the population of axial SpA patients with typical presentation. SPARCC, SPondyloArthritis Research Consortium of Canada; BME, bone marrow edema.

Concerning the SPARCC score for SIJs, the distribution of values is shown in Figure 3 and Supplementary Table S2: the SPARCC score for BME and erosions is significantly higher in the axial SpA patients with typical onset (BME: mean value 20.1 ± 12.28 vs. 6.15 ± 3.21; erosions: 22.16 ± 8.13 vs. 6.60 ± 3.08, respectively). Regarding the ultrasound evaluation of the SIJs, the PW Doppler vascular signal was classified as 0 in one patient (5%), 1 in nine patients (45%), and 2 in ten patients (50%). The mean RI value was 0.49 ± 0.18 (range 0–0.66).

Concerning the correlation between US findings and MR acute inflammatory findings in SIJs, no significant association was found between the PW Doppler scale and the presence/absence of BME (Spearman’s Rank Correlation Coefficient −0.132, *p* = 0.578) or the presence/absence of enthesitis (Spearman’s Rank Correlation Coefficient −0.168, *p* = 0.478). No significant correlation was found between the RI and the presence/absence of BME (Spearman’s Rank Correlation Coefficient −0.289, *p* = 0.217) or the presence/absence of SIJ enthesitis (Spearman’s Rank Correlation Coefficient 0.157, *p* = 0.508).

## Discussion

The occurrence of fever in axial SpA has been described infrequently, and the exact aetiology of this clinical manifestation is unclear. One potential explanation is that the fever could be a manifestation of extra-articular inflammation; alternatively, axial SpA may be part of a more complex clinical picture (6, 7, 20). The clinical approach to autoinflammatory disease involves assessing all clinical elements that may explain the genesis and persistence of systemic inflammation. For this purpose, the creation of the international AIDA registry, dedicated to undifferentiated autoinflammatory diseases, allows for attention to be focused on new clinical and pathological entities in patients with recurrent febrile syndromes of unknown aetiology (10).

A total of 57 patients with chronic low back pain and recurrent febrile syndromes were identified in the AIDA registry: in all these patients, axial SpA could be diagnosed according to the ASAS criteria (imaging arm). The purpose of this study was to analyse the imaging characteristics compared to a population of patients with a typical presentation of axial SpA. Although with a lower prevalence compared to the typical axial SpA population, patients with febrile syndromes also exhibited a notable prevalence of acute inflammatory changes and structural damage to the SIJs, either isolated or combined (9 and 19.2% respectively, vs. 72.8%).

Consistent with the literature data, our study revealed that the most common changes observed in SIJs in axial SpA patients with recurrent fever were subchondral BME (78.9%) and joint erosions (85.9%) (13). The concomitant presence of subchondral BME and joint erosions has been demonstrated to have a 94% specificity in diagnosing sacroiliitis (21). The high prevalence of structural damage of the SIJs in our population is probably related to the relative delay of the diagnosis in the context of a non-specific clinical presentation (11). The inclusion of a panoramic coronal sequence of the hip in our MR protocol allowed the assessment of enthesitic involvement of the pelvis, which was observed with notable prevalence in both patient groups, predominantly at the level of the greater trochanters (73.7%). Pelvic enthesitis occurs in 5–20% of cases of axial SpA, particularly associated with forms of psoriatic arthritis (22, 23). Our data may be related to the study protocol, which includes a panoramic evaluation of the pelvis in all patients and not only those presenting with symptoms, as suggested in the literature: in fact, the study protocol outlined in the literature includes only oblique sequences oriented along the major axis of the SIJs (24).

Active inflammatory involvement of the lumbosacral spine was found, with a high prevalence of inflammatory lesions observed in the posterior elements, including interspinous enthesitis (71.9%) and zygapophyseal joint capsulitis (63.1%). In all cases, spinal inflammatory involvement was associated with signs of active or chronic inflammatory sacroiliitis, a scenario more commonly observed in patients with axial SpA. Hoffstetter et al. report concomitant SIJs and spinal involvement in patients with axial SpA in up to 62.2% of cases, and isolated spinal involvement in 9.8% of cases (25). Vertebral body involvement was less prevalent than posterior element involvement, probably due to an intrinsic limitation of our MR study protocol, which primarily focused on evaluating the pelvis without dedicated sequences for the lumbosacral spine. Consequently, the spine was primarily assessed using panoramic coronal sequences and 3D post-contrast sequences. However, Bochkova et al. reported a higher incidence of inflammatory involvement of the posterior elements of the spine compared to the vertebral body (90.9% vs. 27.2%) (26).

Regarding the use of CM, no significant diagnostic advantage has been demonstrated. In the literature, it is reported that CM sequences are more sensitive than fat-saturated fluid-sensitive sequences in evaluating acute inflammatory lesions of SIJs and are beneficial to ensure maximum diagnostic confidence when examining patients with early sacroiliitis (27–29). However, fat-saturated fluid-sensitive sequences alone are sufficient for establishing a reliable diagnosis (27, 30).

In a subset of patients with axial SpA group with recurrent fevers (20/57), US assessment of the SIJs was performed; however, no significant correlation with MRI findings was observed. US evaluation of the SIJs remains an unvalidated technique with limited clinical utility in axial SpA patients (30).

This study cannot elucidate the role of fever in the pathogenesis of SIJ and spinal lesions observed in axial SpA patients with recurrent fever. However, the different prevalence of inflammatory and structural lesions between patients with axial SpA with recurrent fever and those with typical onset could be explained by the difference in disease duration between the two groups (5.21 vs. 11.8 years).

The additional main limitation of our study is its retrospective and descriptive nature, as well as the restricted cohort of patients included in the analysis. Although the ASAS criteria were developed for patients up to 45 years of age, they were also applied to patients over 45, as no other criteria could be used for preradiographic forms. These forms, having escaped early diagnosis and not progressing to a radiographic stage, were identified after the age of 45.

In conclusion, a high prevalence of active and chronic inflammatory changes in the SIJs, enthesitic involvement of the pelvis, and inflammatory changes in the lumbar spine were observed in patients with axial SpA presenting with recurrent febrile syndromes. Axial SpA should be considered among the possible differential diagnoses in patients presenting recurrent febrile syndromes of unknown aetiology and inflammatory back pain, as demonstrated in our patient cohort. Enthesitis of the pubic symphysis, vertebral body corner sclerosis, zygapophyseal capsulitis, and interspinous enthesitis were the only radiologic lesions that showed a significant difference in frequency between axial SpA patients with and without fever attacks.

## Data Availability

The original contributions presented in the study are included in the article/Supplementary material, further inquiries can be directed to the corresponding authors.
