# Supplementary material for: Imaging findings in patients with axial spondyloarthritis presenting with recurrent fever attacks: data from the international AIDA network spondyloarthritis registry
Source: Front Med (Lausanne). 2025 Sep 15;12:1662890. doi: 10.3389/fmed.2025.1662890 (PMC12477155; doi:10.3389/fmed.2025.1662890)
Supplement: Supplementary file 1 [file Table_1.DOCX]

**Supplementary Materials**

**Table S1** – Magnetic Resonance protocol.

|  | **TR**  (ms) | **TE**  (ms) | **TI**  (ms) | **NEX** | **Bandwidth** (hKZ) | **SS/Overlap** (mm) | **FOV**  (cm) | **Acquisition plane** |
| --- | --- | --- | --- | --- | --- | --- | --- | --- |
| STIR FRFSE | 5432 | 63 | 140 | 2 | ± 41.7 | 4/0.5 | 34x42 | Coronal hip full FOV |
| T1-weighted FSE | 398 | 9 | - | 2 | - | 4/0.4 | 24x24 | Coronal oblique to SIJs |
| STIR FRFSE | 3824 | 67 | - | 2 | ± 31.2 | 4/0.4 | 24x24 | Coronal and axial oblique to SIJs |
| 3D T1-weighted/Gd FSPGR | 6.7 | 2.3 | 20 | 2 | ± 41.7 | 3.2/1.6 | 26x26 | Coronal oblique to SIJs |
| 3D T1-weighted/Gd  FSPGR | 4.1 | 1.3 | 16 | 1.5 | ± 62.5 | 1.6/0.9 | 40x40 | Coronal hip full FOV |
| **FS** Fat-Saturated **FSE** Fast Spin Echo **FRFSE** Fast Recovery Fast Spin-Echo **Gd** before and after intravenous contrast injection **FSPGR** SPoiled GRadient-Echo with Spectral Inversion at Lipid **FOV** Field of view | | | | | | | | |

**Table S2** – SPondyloArthritis Research Consortium of Canada (SPARCC) score for acute and chronic inflammatory changes of the sacroiliac joints (SIJs) at Magnetic Resonance examination.

|  | **Axial SpA with recurrent fever** | **Typical onset Axial SpA without recurrent fever** | **Statistical significance** |
| --- | --- | --- | --- |
| BME  *Mean ± SD*  *Median*  *Min-Max value* | 6.15±3.21  5.50  2-17 | 20.1±12.28  16.50  6-56 | 0.01 |
| Subchondral Sclerosis  *Mean ± SD*  *Median*  *Min-Max value* | 6.21±3.50  5.00  2-15 | 9.08±4.70  9.00  2-19 | 0.019 |
| Fat Metaplasia  *Mean ± SD*  *Median*  *Min-Max value* | 8.92±4.15  8.00  3-18 | 13.93±7.04  14.00  2-30 | 0.01 |
| Erosions  *Mean ± SD*  *Median*  *Min-Max value* | 6.60±3.08  6.50  2-15 | 22.16±8.13  20.00  11-38 | 0.01 |
| BME: Bone Marrow Edema; SpA: spondyloarthritis; SD: standard deviation. | | | |
